# Arthroscopic proficiency: methods in evaluating competency

**DOI:** 10.1186/1472-6920-13-61

**Published:** 2013-05-01

**Authors:** Justin L Hodgins, Christian Veillette

**Affiliations:** 1 Division of Orthopaedics, Toronto Western Hospital, Toronto, Canada; 2 University of Toronto Sports Medicine Program, Women’s College Hospital, Toronto, Canada

**Keywords:** Arthroscopy, Competency, Surgical training, Task performance

## Abstract

**Background:**

The current paradigm of arthroscopic training lacks objective evaluation of technical ability and its adequacy is concerning given the accelerating complexity of the field. To combat insufficiencies, emphasis is shifting towards skill acquisition outside the operating room and sophisticated assessment tools. We reviewed (1) the validity of cadaver and surgical simulation in arthroscopic training, (2) the role of psychomotor analysis and arthroscopic technical ability, (3) what validated assessment tools are available to evaluate technical competency, and (4) the quantification of arthroscopic proficiency.

**Methods:**

The Medline and Embase databases were searched for published articles in the English literature pertaining to arthroscopic competence, arthroscopic assessment and evaluation and objective measures of arthroscopic technical skill. Abstracts were independently evaluated and exclusion criteria included articles outside the scope of knee and shoulder arthroscopy as well as original articles about specific therapies, outcomes and diagnoses leaving 52 articles citied in this review.

**Results:**

Simulated arthroscopic environments exhibit high levels of internal validity and consistency for simple arthroscopic tasks, however the ability to transfer complex skills to the operating room has not yet been established. Instrument and force trajectory data can discriminate between technical ability for basic arthroscopic parameters and may serve as useful adjuncts to more comprehensive techniques. There is a need for arthroscopic assessment tools for standardized evaluation and objective feedback of technical skills, yet few comprehensive instruments exist, especially for the shoulder. Opinion on the required arthroscopic experience to obtain proficiency remains guarded and few governing bodies specify absolute quantities.

**Conclusions:**

Further validation is required to demonstrate the transfer of complex arthroscopic skills from simulated environments to the operating room and provide objective parameters to base evaluation. There is a deficiency of validated assessment tools for technical competencies and little consensus of what constitutes a sufficient case volume within the arthroscopy community.

## Background

The evolution of diagnostic and therapeutic techniques has made arthroscopy one of the most commonly performed orthopaedic procedures [1]. Despite its prevalence, arthroscopy is technically demanding requiring visual-spatial coordination to manipulate instruments while interpreting three-dimensional structures as two-dimensional images. These skills are traditionally acquired through the apprenticeship model of step-wise involvement in the operating room, but the process is inefficient in terms of time and cost and associated with iatrogenic injury to the patient [2-5]. With the increasing complexity of arthroscopic procedures and the implementation of work-hour restrictions, the adequacy of arthroscopic training during residency has become a concern [6,7].

To combat insufficiencies, emphasis in post-graduate training is shifting towards specific skill acquisition and the achievement of technical competencies [8]; this is the rationale behind improving arthroscopic skill development outside of the operating room. The advent of surgical simulation, psychomotor conditioning and the cadaveric bioskills laboratory as useful training adjuncts is encouraging [4,5,9-15]. Despite these efforts, evidence suggests that residents feel less prepared in arthroscopic training compared to open procedures and a substantial number of procedures may be required to become proficient [16-18]. The necessary operative experience and instruction to attain competency is uncertain. Currently, the Residency Review Committee for the Accreditation Council of Graduate Medical Education (ACGME) requires only a record of completed arthroscopic procedures and does not specify what constitutes a sufficient case volume [19].

As pressures for training standardization and certification mount, there remains no objective testing to evaluate arthroscopic competency at the end of an orthopaedic residency [20-22]. The identification of effective arthroscopic teaching methods and evaluation tools is first necessary to determine what constitutes sufficient training. There is need for comprehensive assessment using true indicators of competence as consensus on defining competence and quantifying arthroscopic proficiency has not been established.

In this article, we reviewed knee and shoulder arthroscopy with respects to (1) the validity of cadaveric models and surgical simulation in arthroscopic training, (2) the role of psychomotor analysis and arthroscopic technical ability, (3) what validated assessment tools are available to evaluate technical competency, and (4) how arthroscopic proficiency is quantified by the regulating bodies and orthopaedic societies.

## Methods

**Figure 1:**
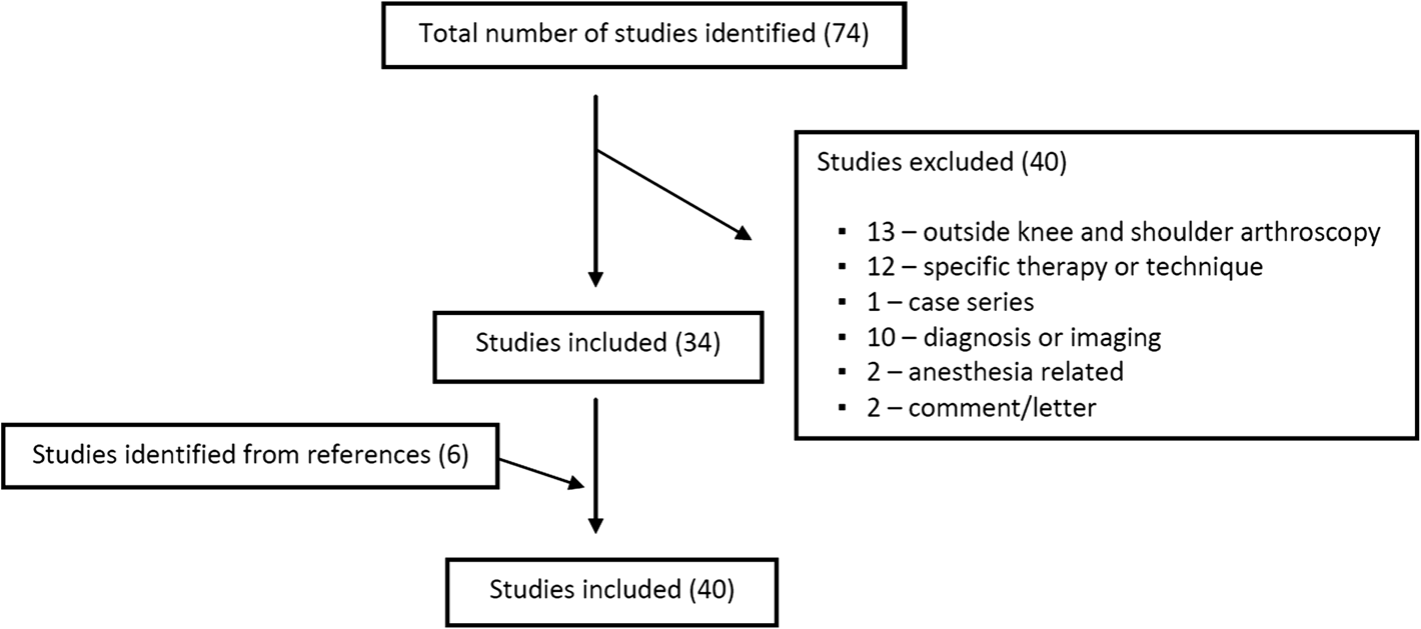
MedLine database search results (34 + 6 of 74 studies included).

**Figure 2:**
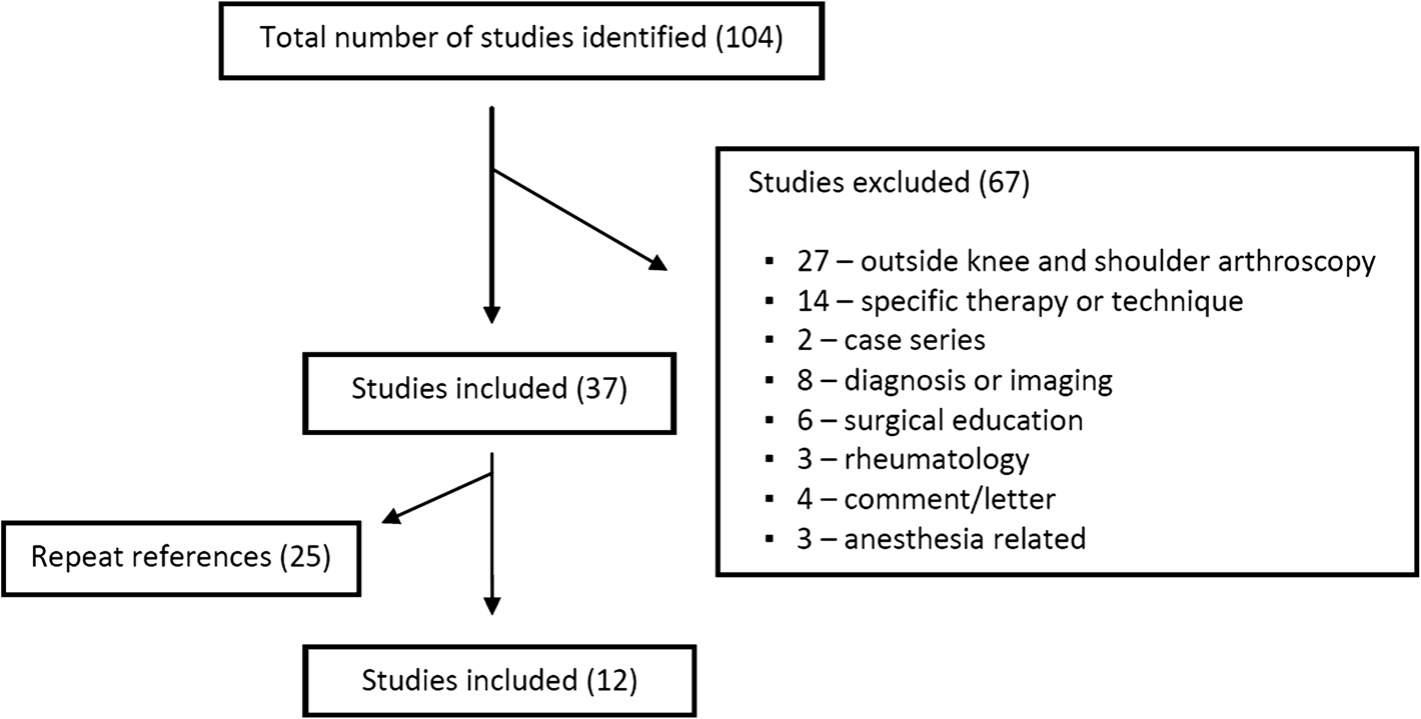
EMBASE database search results (12 of 104 studies included).

A comprehensive search of the Ovid MedLine (Figure 1) and EMBASE (Figure 2) databases published in the English literature was performed. Search terms were altered for each database according to its method of subheading mapping. The search results and number of studies found at each stage are listed below:

Ovid MedLine: 1996 to February Week 2, 2013

1. exp Clinical Competence: 47 976

2. exp Learning curve: 4 588

3. exp Task Performance and Analysis: 18 681

4. 1 OR 2 OR 3: 69 539

5. exp Arthroscopy: 11 490

6. 4 AND 5: 79

7. Limit 6 to English language and Humans: 74

EMBASE: 1980 to February Week 2, 2012

1. exp competence: 82 920

2. exp surgical training: 10 573

3. exp task performance: 93 865

4. exp learning curve: 1 862

5. 1 or 2 or 3 or 4: 185 919

6. exp arthroscopy: 18 210

7. 5 and 6: 132

8. Limit 6 to English language and Humans: 104

Two reviewers (JLH, CV) independently evaluated the abstracts of the search results. Studies selected underwent fulltext reviews and were original research or review articles pertaining to (1) arthroscopic competence, (2) arthroscopic assessment and evaluation, and/or (3) objective measures of arthroscopic technical skill. Exclusion criteria included article topics (1) outside the scope of knee and shoulder arthroscopy, (2) therapeutic treatments and outcomes, (3) diagnostic imaging, and (4) case series. Studies were excluded only if there was mutual agreement between the two reviewers. Relevant references from each of the remaining articles were examined for inclusion. Articles were then cross-referenced to discard repeated references, leaving 52 orthopaedic articles cited in this review.

## Results

### Cadaveric training models and surgical simulation

Advancing technical complexity taught within reduced work-hour training programs has driven the need for alternative strategies in arthroscopic skill development. Traditionally, the cadaver specimen in the bioskills laboratory has remained the highest fidelity model [23]. Few would contest the likeness of the human cadaveric specimen to reproduce arthroscopy conditions or the value of positional and tactile feedback using instrumentation in this environment. The use of fresh cadaveric specimens as the primary teaching platform in instructional courses for board certified surgeons supports this claim. The educational benefits of managing the nuances of arthroscopic equipment and troubleshooting problems with fluid management, the light source, and shavers should also not be underestimated [17]. In addition, when arthroscopic trained surgeons were polled on training methods contributing to self-perceived proficiency in all-arthroscopic rotator cuff repair, practice on cadaveric models was third, second only to fellowship training and hands-on courses [24].

Financial considerations as well as specimen availability limit formal arthroscopic training on cadavers within most orthopaedic program curricula [4]. The cost of acquiring specimens combined with the inherent costs of maintaining the equipment and personnel of a bioskills laboratory are difficult to quantify and usually depend on industry support and/or sponsorship. There are also concerns regarding uniformity between specimens with variability in both anatomy and internal pathology [4].

To avoid these obstacles, the concept of computer-based simulation for arthroscopic training and skill acquisition has emerged. Embraced by the aviation industry, simulators remain a core competency in pilot training and credentialing [11]. The development of less-expensive high performance computers combined with advances in graphical and force-feedback technology (haptics) has accelerated this movement. Proposed simulators would allow for the quantitative assessment of technical ability performed within the confines of a safe and controlled environment. Advantages include the absence of time constraints or supervising faculty, uniform training scenarios of adjustable complexity and pathology as well as substantial saving from costly disposable equipment and training time within the operating room [15,23].

The use of laparoscopic and endoscopic simulators has been incorporated into many training programs, as the validity of such models has been previously established [25-30]. A systematic review of randomized controlled trials of laparoscopic simulators reported improved task performance by trainees and a greater reduction in operating time, error and unnecessary movements as compared to standard laparoscopic training [31].

In contrast to laparoscopy, the focus of the arthroscopic literature has been the validation of particular simulators as this technology continues to be refined (Table 1). The notion of construct validity, the correlation between arthroscopy expertise and simulator performance, has been demonstrated within the shoulder [5] and knee models [12,13,32,33]. Alternatively, transfer validity is the correlation between performance in the simulator and that in a cadaver model or actual surgical procedures.

**Table 1:** Arthroscopic simulation studies

**Study**	**Validity**	**Outcome**	**Conclusion**
McCarthy et al. [12]	Construct (knee)	Time to task completion; number of arthroscope and probe collisions	Increased surgical experience associated with reduced probe collisions and time to completion
Smith et al. [34]	Construct (shoulder)	Number of task errors; number of probe and dangerous collisions and path length ratio	Task performance able to discriminate arthroscopy experience; lower time to completion and number of collisions for orthopaedic surgeons
Sherman et al. [33]	Construct (knee)	Mean score for structures indentified; time to task completion; composite score	Differences in scoring performance between individual trainees identified
Pedowitz et al. [11]	Construct (shoulder)	Time to task completion; distance probe traveled; number of probe collisions	Improved arthroscopic performance with increasing arthroscopic experience
Bliss et al. [35]	Construct (knee)	Number of anatomical landmarks identified; manipulation score out of 100	Simulator is effective teaching method for learning basic anatomy and manipulation skills
Gomoll et al. [5]	Construct (shoulder)	Time to task completion; distance traveled by probe; speed of probe; number of probe collisions	Improved simulator performance with surgical experience for all parameters
Gomoll et al. [10]	Construct (shoulder)	Time to task completion; distance traveled by probe; speed of probe; number of probe collisions	Increased surgical experience over 2 year period associated with improved simulator performance
Howells et al. [13]	Transfer (knee)	OCAP; OSATS	Trainees with simulator training have improved performance in operating room compared to untrained control
Tashiro et al. [32]	Construct (knee)	Path length of arthroscopic scissors; path length of arthroscopic probe	Simulator scoring and time to completion able to discriminate level of surgical skill and experience
Martin et al. [15]	Transfer (shoulder)	Time to completion of arthroscopic task	Strong correlation between arthroscopic task performance in simulator and cadaveric models
Martin et al. [36]	Transfer (shoulder)	Time to completion of arthroscopic task	Simulator performance correlates with resident arthroscopic skill and experience

Abbreviations: OCAP, Orthopaedic Competence Assessment Project; OSATS, Objective Structured Assessment of Technical Skill.

Knee simulators have been shown to reliably distinguish between novice and expert arthroscopists [12,32] and demonstrate the learning potential of identifying anatomical landmarks and triangulation skills [35]. There is only a single study demonstrating the transfer validity of arthroscopic skills to the operating room for diagnostic knee arthroscopy [13]. However, there was no true control group having only compared simulator training versus no training.

Outcome measures that were able to discriminate skill level and expertise in shoulder simulators include; time to completion of tasks, distance and path traveled by probe and the number of probe collisions [5,11,15,34]. A follow-up study conducted at the 3-year period showed significantly improved simulator performance after an increase in arthroscopic experience [10]. A positive correlation of arthroscopic task performance between simulator and cadaveric models has also been observed in shoulder arthroscopy [15]. A subsequent investigation demonstrated a significant relationship between the performance of basic arthroscopic tasks in a simulator model and resident arthroscopic experience, supporting the use of simulators as beneficial educational tools to improve arthroscopic skills [36].

Technological advances have made the potential widespread use of simulators more affordable, but additional hurdles exist. The availability of content experts, mainly surgeons that can provide domain-specific surgical knowledge to allow developers to generate realistic simulations is a limiting factor [9]. Further understanding of the psychomotor and cognitive components of the surgical process is still necessary for its translation into the virtual world.

### Psychomotor analysis and arthroscopic technical ability

The technical capabilities of the surgeon continue to expand as minimally invasive surgery evolves. This is especially true in arthroscopy, where triangulation and visual-spatial coordination are essential for task completion. This has been accompanied by growing interest in methods of evaluation to further refine psychomotor skills. Measuring a sensitive technical parameter could provide an objective marker of arthroscopic technical ability used to validate simulators and evaluate trainee performance [37]. These parameters can be characterized into those measuring force patterns (haptics) and those focused on trajectory data and motion analysis (Table 2).

**Table 2:** Arthroscopic studies using psychomotor analysis

**Study**	**Design**	**Outcome**	**Conclusion**
Gomoll et al. [5]	Trajectory patterns (shoulder)	Time to task completion; distance traveled by probe; speed of probe, number of probe collisions	Improved simulator performance with surgical experience for all parameters
Howells et al. [37]	Trajectory patterns (shoulder)	Time to task completion; probe path length; number of movements	Increased surgical experience associated with improved economy of movements
Chami et al. [38]	Force/trajectory patterns (knee)	Torque magnitudes during arthroscopic tasks; time to completion, navigation paths	Improved efficiency and reduced force magnitudes with increasing arthroscopic experience
Tashiro et al. [32]	Force/trajectory patterns (knee)	Time to completion; instrument trajectory; surgical force	Simulator scoring and time to completion able to discriminate level of surgical skill and experience
Tuijthof et al. [39]	Force (knee)	Absolute maximum probing force (AMPF)	Safe AMPF identified as < 8.5N, inherent differences between novice and expert skill
Alvand et al. [40]	Visual parameters/motion analysis	Prevalence of instrument loss, triangulation time, prevalence of lookdowns	Simulator scoring able to discriminate between novice, resident and expert skill levels

Analysis of force sensors has been reported as a valuable method to assess interference between surgical tools and tissue in endoscopic sinus surgery and laparoscopic surgical training [41,42]. In arthroscopy, excessive force applied through instruments may result in iatrogenic damage to the patient, often as damage to articular cartilage [11,23,43]. Therefore, measurements of force may provide an objective means of evaluating tactile surgical performance. Assessment of force torque signatures have been shown to correlate with level of arthroscopic experience in the knee, where expert surgeons had fewer collision incidences, greater variety of force magnitudes and superior efficiency [38]. The use of excessive and unnecessary force patterns by trainees was also demonstrated in a knee simulator when compared to that of experienced surgeons [32]. However, distinguishing harmful from harmless contact in tissue manipulation and dissection can be challenging and these studies were small and often lacking a complete assessment of each area of the knee. The concept of absolute maximum probing force (AMPF) during menisci manipulation has been introduced and significant differences between the expert and novice arthroscopists have been identified [39].

Electromagnetic motion tracking systems have been employed to plot instrument tip trajectory as an objective evaluation tool. The validity of motion analysis to assess surgical skills in terms of precision and economy of movement has been shown within the laparoscopy literature [44,45]. In knee arthroscopic simulators, level of expertise has been associated with reduced probe path traveled and number of movements and improved economy of movements [37]. Similarly, the path length of the probe and scissors was substantially shorter and probe velocity faster in more experienced surgeons when performing partial meniscectomy in knee models [32]. These finding have also been demonstrated in virtual reality simulators of the shoulder where probe path length was shorter for specialists and probe velocity was nearly double that of novices [5]. Reduced traveled probe distance has been suggested to correlate with smoothness of scope manipulation during shoulder joint inspection and probing tasks. Yet, motion analysis investigations have only been performed within a simulated environment and only involving basic arthroscopic tasks. It is unclear if improved efficiency of movements in these models translates into improved performance within the operating theatre.

In addition to force and motion analysis, simple visual parameters have been described as an objective method for evaluating technical skill [40]. The prevalence of instrument loss, lookdowns and triangulation time is able to discriminate novice, resident and expert skill levels in a knee simulator.

### Validated assessment tools

The current paradigm of arthroscopic training relies on the apprenticeship model where residents are evaluated by a precepting surgeon as their level of involvement is subsequently increased. The subjectivity of this method has been criticized and shown to not necessarily reflect the actual level of skill [23,43]. This assessment is not based on a pre-determined level of performance, but rather on global assessment by the precepting surgeon partly determined by that surgeon’s experience and spectrum of patients within their practice [9]. Ideally, an assessment tool should be feasible and practical while remaining as objective as possible [37,46].

The implementation of various procedure-specific checklists [47-49] and global rating scales [50-52] has been well described in other surgical disciplines and the Objective Structured Assessment of Technical Skill (OSATS) is the most widely accepted “gold standard” for objective skills assessment [53]. Yet, evidence suggests that these methods are valid for feedback and measuring progress of training rather than examination or credentialing [54].

Within orthopaedics, particularly arthroscopy, research into objective evaluation techniques is more limited (Table 3). The Basic Arthroscopic Knee Scoring System (Additional file 1: Appendix 1-A, 1-B) is a two-part assessment that has been validated in cadaver specimens [17]. It is composed of a task-specific checklist (TSCL) measuring what components of a diagnostic arthroscopy and partial meniscectomy a subject completes and a global rating scale (GRS) documenting how well these tasks are completed. Both the TSCL and then GRS have been shown to differentiate levels of arthroscopic skill and objectively evaluate basic arthroscopic proficiency in the bioskills laboratory [17].

**Table 3:** Validated arthroscopic assessment tools

**Study**	**Tool**	**Description**	**Conclusion**
Howells et al. [13]	Modified Orthopaedic Competence Assessment Project (knee)	Intra-operative 14 point arthroscopic checklist and OSATS GRS	Tool demonstrated improved performance in operating room for simulator trained individuals compared to untrained control
Insel et al. [17]	The Basic Arthroscopic Knee Scoring System (knee)	Combined TSCL and GRS for diagnostic knee arthroscopy and partial meniscectomy	System able to discriminate between individuals with different levels of arthroscopic experience
British Orthopaedic Specialist Advisory Committee [55]	Orthopaedic Competence Assessment Project (knee)	Intra-operative 14 point arthroscopic checklist	--
			Has not been subjected to validity testing
Elliott et al. [56]	Arthroscopic Skills Assessment Form (knee)	100-point score, 75 for structure identification, 25 for time to completion and deductions for cartilage injury	Can distinguish between the novice, experienced and expert arthroscopists in the cadaver knee
Shantz et al. [57]	The Objective Assessment of Arthroscopic Skills (OAAS) (knee)	Global skills domains with 5 skill-level options combined with 13 point anatomical area checklist	Discriminates between various skill level of training, high internal consistency and test-retest reliability

Abbreviations: TSCL, Task Specific Check List; GRS, Global Rating Scale; OSATS, Objective Structured Assessment of Technical Skill.

The Orthopaedic Competence Assessment Project, developed by the British Orthopaedic Specialist Advisory Committee, is part of the competency-based training structure implemented by the surgical royal colleges in the United Kingdom [55,58]. It consists of an intra-operative technique section comprised of 14 criteria, but has not been subjected to independent testing. However, a modification of this procedural-based assessment (Additional file 2: Appendix 2-A) combined with an OSATS global rating scale (Additional file 2: Appendix 2-B) was developed to evaluate the transfer validity of a simulator in diagnostic knee arthroscopy [13]. Although improved performance in the simulator-trained group was demonstrated, the only comparison was an untrained group of individuals.

Recently, more comprehensive knee scoring systems have been introduced. The Arthroscopic Skills Assessment Form is a 100-point tool used to objectively evaluate diagnostic knee arthroscopy assigning points for correctly identifying structures and time to completion as well as point deductions for iatrogenic cartilage injury [56]. It was able to distinguish between the novice, experienced and expert arthroscopists in the cadaver knee model. The Objective Assessment of Arthroscopic Skills (OAAS) instrument consists of multiple skill domains each rated on an expertise-based scale with 5 skill-level options [57]. When combined with an anatomical checklist, the OAAS discriminated between skills levels of various levels of training with excellent internal consistency and test-retest reliability.

### Quantifying arthroscopic proficiency

Despite being amongst the most commonly performed procedures by orthopaedic surgeons, consensus on what constitutes arthroscopic competence and the number of procedures to attain it remains uncertain [16,18]. This is compounded by increasing technical sophistication of procedures and the demand for accountability and satisfactory outcomes by patients [59]. Competency in arthroscopy typically develops during completion of a residency curriculum as defined by the Residency Review Committee for the ACGME, but there is no suggestion for a recommended case volume of procedures [19]. Certification examinations test for proficiency in content comprehension and decision-making capabilities, yet there is no objective testing to evaluate arthroscopic technical competencies at the end of residency [20-22].

Objective data regarding competence in arthroscopy is sparse and guidelines specifying achievement and maintenance of competence are vague. The Arthroscopy Association of North America (AANA) does not quantify competence, but only requires that 50 arthroscopic cases be performed annually to maintain active membership [60]. However, the AANA does acknowledge that completion of an orthopaedic residency does not guarantee competence in arthroscopy and that privileges should be granted by the regulating bodies of individual hospitals and should consist of an observational period for direct skill assessment [61]. The American Board of Orthopedic Surgery (ABOS) requires a one-year accredited ACGME sports medicine fellowship and at least 75 arthroscopy cases to be eligible for subspecialty certification in sports medicine [62].

Considerable variation exists when attempting to assign a numerical value for arthroscopic competency in the literature. A survey of U.S. orthopaedic department chairs and sports medicine fellowship directors identified substantial variability in the number of repetitions to become proficient in arthroscopy [18]. For instance, the average number for diagnostic arthroscopy of the knee was 45 with suggested repetitions ranging from 8 to 250. There was also a tendency for physicians who perform little or no arthroscopy to underestimate the experience needed for proficiency. This finding was confirmed in a similar survey performed in Europe amongst orthopaedic residents and attending staff as well as the trend for residents to overestimate the average number of cases required for competency [63]. Here, a mean of 120 procedures was estimated by residents for arthroscopic ACL reconstruction compared to 90 by staff physicians.

## Discussion

The current paradigm of arthroscopic training combined with increased complexity and frequency of procedures has led to questioning of its adequacy. This review examines arthroscopic skill development constructs, objective assessment tools, and guidelines regarding arthroscopic competencies.

Cadaver specimens are a highly regarded training modality for arthroscopic technical skill development and remain the gold standard for training outside of the operating room. Despite concerns regarding pathology consistency in specimens, cost and availability are the primary constraints to their widespread use [4]. The introduction of synthetic and plastic bone models have the advantage of anatomical reproducibility without maintenance or ethical issues, but have been criticized for a lack of face validity [64].

Computer-based simulators are moving from the experimental stages with established construct validity in knee and shoulder arthroscopy [5,12,13,32,33]. Improved, less-expensive computer hardware has made the technology more readily available fueling the investigation of their training potential in arthroscopic task performance. These studies have high levels of internal validity and consistency, although most involve only basic arthroscopic skills, such as orientation and triangulation or only demonstrate improved performance in individuals with no previous arthroscopic experience [65]. Likewise, most validated simulators are only sensitive enough to discriminate between expert and novice skill levels [66]. The ability to detect smaller, yet clinically significant differences between intermediate skill levels is required to establish benchmarks and provide objective feedback to the training population of residents.

Studies focusing on complex tasks, such as simulated arthroscopic meniscal repair have exhibited learning curves and skill retention, but whether this translates into improved performance within the operating room has not yet been established [67]. Two systematic reviews have failed to identify sufficient evidence of transfer validity within the arthroscopic literature [66,68]. This is also complicated by the heterogenicity of existing simulators being subjected to validity testing [66]. Further high-quality studies are required before the widespread acceptance of these tools into mainstream arthroscopic training programs. This includes the establishment of skill-sensitive simulators with standardized validity protocols that consistently translate into improved technical performance in the operating room.

Surgical dexterity focusing on parameters extracted from instrument force and trajectory data may provide an alternate means of objective evaluation. A greater variety of force signatures and a reduction in excessive and unnecessary probe forces by expert compared to novice arthroscopists has been demonstrated within the knee model [32,38]. The use of motion analysis to discriminate between levels of arthroscopic experience in terms of economy of instrument movement and probe velocity has been validated in both knee and shoulder simulation [5,32,37,44,45]. As with arthroscopic simulations and cadavers, psychomotor analysis has only been validated when performing basic arthroscopic tasks predominantly in simulated environments and does not provide a comprehensive assessment of performance. However, these parameters may serve as potential adjuncts to traditional means of evaluation and have a role in selecting individuals for surgical disciplines based on innate arthroscopic ability. Significant differences in multiple motion analysis parameters was shown in medical students who failed to achieve competence despite sustained practice when completing an arthroscopic task in knee and shoulder models compared to those who achieved competence [69,70].

Traditional arthroscopic training during residency lacks a standardized, objective evaluation system. The existing preceptor-dependent model is subjective and inefficient in terms of time and cost [2,11,23,43]. There are a limited number of studies dedicated to the development and validation of comprehensive assessments of arthroscopic technical skills. The Basic Arthroscopic Knee Scoring System can discriminate between different levels of arthroscopic expertise, but has only been validated in cadaver specimens and when performing basic arthroscopic tasks [17]. Similarly, modifications to the intra-operative technique guidelines of the Orthopaedic Competence Assessment Project and the addition of a tailed OSATS scale were applied to assess diagnostic knee arthroscopy [13,55]. The project demonstrated transfer validity to the operating theatre, but simulator-trained subjects were compared to those with no training at all. More recently, comprehensive global assessment instruments such as the Arthroscopic Skills Assessment Form and the OAAS instrument have been shown to discriminate between various skills levels of training and provide additional domains of evaluation with high levels of internal consistency. Objective assessment tools are essential for effective and efficient learning as deficiencies in performance are difficult to correct without objective feedback [17]. Yet, few such instruments exist within the arthroscopic literature, particularly for the shoulder.

The case volume required to be considered competent in a specific arthroscopic procedure remains uncertain [16,18]. The Residency Review Committee for the ACGME only requires a log of accumulated arthroscopic procedures and no objective evaluation of technical skills exists at the completion of residency [20-22]. The AANA does not designate a numerical value to be proficient in arthroscopy and concedes that residency training alone does not guarantee competency [60,61]. Consensus on what constitutes a sufficient repetition of a procedure varies considerably when surveying the orthopaedic community and there is a tendency for underestimation by those who perform arthroscopy sparingly [18,63]. There is suggestion that proficiency in arthroscopy is only attained after completing a case range equivalent to that of a sports medicine fellowship [17]. Few would contest that there is no substitute for experience, but how much is needed and when proficiency is achieved remains unknown.

## Conclusion

There is uncertainty concerning the adequacy of arthroscopic training and the best means to achieve technical competencies. Skill acquisition utilizing surgical simulation requires further demonstration of transfer validity and the application of complex arthroscopic tasks in these environments. Valid assessment tools evaluating technical performance are required to establish objective parameters in arthroscopic training to generate standardized benchmarks of competency and ultimately improve technical proficiency.

## Competing interests

Each author certifies that he or she has no commercial associations (eg, consultancies, stock ownership, equity interest, patent/licensing arrangements, etc) that might pose a conflict of interest in connection with the submitted article.

## Authors’ contributions

JLH Conceived the manuscript, conducted the literary search and helped draft the manuscript. CV Participated in the design of the manuscript, helped draft, revise and critically appraise manuscript content. Both authors read and approved the final manuscript.

## Pre-publication history

The pre-publication history for this paper can be accessed here:

http://www.biomedcentral.com/1472-6920/13/61/prepub

## Supplementary Material

Additional file 1 **Appendix 1-A.** The Basic Arthroscopic Knee Scoring System: Global Rating Scale [17]. **Appendix 1-B:** The Basic Arthroscopic Knee Scoring System: Task-Specific Checklist [17]. Click here for file

Additional file 2 **Appendix 2-A.** Intra-operative technique section of the Orthopaedic Competence Assessment Project arthroscopy procedure-based assessment. **Appendix 2-B:** Objective Structured Assessment of Technical Skill (OSATS) Global Rating Scale. Click here for file
