# Supplementary material for: Arthroscopic proficiency: methods in evaluating competency
Source: BMC Med Educ. 2013 May 1;13:61. doi: 10.1186/1472-6920-13-61 (PMC3643847; doi:10.1186/1472-6920-13-61)
Supplement: Additional file 1 — Appendix 1-A. The Basic Arthroscopic Knee Scoring System: Global Rating Scale [17]. Appendix 1-B: The Basic Arthroscopic Knee Scoring System: Task-Specific Checklist [17]. [file 1472-6920-13-61-S1.docx]

| Bio-skills Lab: Knee Arthroscopy | Subject Number: |
| --- | --- |
| Global Rating Scale (GRS) | Test Date:  ______/______/______ |


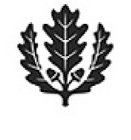


**Study**

**Form**

**Study**

**Form**

| **Please circle the number (1-5) that best describes the subject** |
| --- |
| **1. Dissection**  1 - Appeared excessively hesitant, caused trauma to tissues, did not dissect into correct anatomical plane  2 -  3 - Controlled and safe dissection into correct anatomical plane, caused minimal trauma to tissues  4 -  5 - Superior and atraumatic dissection into the correct anatomical plane |
| **2. Instrument Handling**  1 - Repeatedly makes tentative or awkward movements with instruments  2 -  3 - Competent use of instruments, although occasionally appeared stiff or awkward  4 -  5 - Fluid moves with instruments and no awkwardness |
| **3. Depth Perception**  1 - Constantly overshoots target, slow to correct  2 -  3 - Some overshooting or missing of target  4 -  5 - Accurately directs instruments in the correct plane to target |
| **4. Bimanual Dexterity**  1 - Noticeably awkward with non-dominant hand, poor coordination between hands  2 -  3 - Uses both hands but does not maximize interaction between hands  4 -  5 - Expertly uses both hands in complementary manner to provide optimum performance |
| **5. Flow of Operation and Forward Planning**  1 - Appeared excessively hesitant, caused trauma to tissues, did not dissect into correct anatomical plane  2 -  3 - Controlled and safe dissection into correct anatomical plane, caused minimal trauma to tissues  4 -  5 - Superior and atraumatic dissection into the correct anatomical plane |
| **6. Knowledge of Instruments**  1 - Frequently stopped operating or needed to discuss next move  2 -  3 - Demonstrated ability for forward planning with steady progression of operative procedure  4 -  5 - Obviously familiar with the instruments required and their names |
| **7. Efficiency**  1 - Many unnecessary, inefficient movements. Constantly changing focus or persisting without progress  2 -  3 - Slow, but planned movements are reasonably organized with few unnecessary or repetitive movements  4 -  5 - Confident, clear economy of movement and maximum efficiency |
| **8. Knowledge of Specific Procedure**  1 - Deficient knowledge, needed specific instruction at most operative steps  2 -  3 - Knew all important aspects of the operation  4 -  5 - Demonstrated familiarity with all aspects of the operation |
| **9. Autonomy**  1 - Unable to complete the entire task, even with verbal guidance  2 -  3 - Able to complete the task safely with moderate guidance  4 -  5 - Able to complete task independently without prompting |
| **10. Quality of final product**  1 - Very poor  2 -  3 - Competent  4 -  5 - Clearly superior |

**APPENDIX 1-A**: The Basic Arthroscopic Knee Scoring System: Global Rating Scale [17]

| Bio-skills Lab: Knee Arthroscopy | Subject Number: |
| --- | --- |
| Task-Specific Checklist (TSCL) | Test Date:  ______/______/______ |


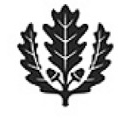


**Study**

**Form**

| Diagnostic Arthroscopy | YES | NO |
| --- | --- | --- |
| 1. Inspection of suprapatellar pouch | □ | □ |
| 2. Inspection of patellofemoral compartment | □ | □ |
| 3. Inspection of lateral gutter | □ | □ |
| 4. Inspection of medial gutter | □ | □ |
| 5. Inspection of medial compartment including anterior and posterior horns | □ | □ |
| 6. Inspection of intercondylar notch including ACL & PCL | □ | □ |
| 7. Inspection of lateral compartment including anterior and posterior horn | □ | □ |

| Partial Meniscectomy | YES | NO |
| --- | --- | --- |
| 1. Appropriate amount of meniscus removed | □ | □ |
| 2. Appropriate use of meniscal biters (basket forceps) and mechanized shavers | □ | □ |
| 3. Contouring of peripheral rim of remaining meniscus | □ | □ |
|  | /10 |  |

**APPENDIX 1-B**: The Basic Arthroscopic Knee Scoring System: Task-Specific Checklist [17]
