# Supplementary material for: Arthroscopic proficiency: methods in evaluating competency
Source: BMC Med Educ. 2013 May 1;13:61. doi: 10.1186/1472-6920-13-61 (PMC3643847; doi:10.1186/1472-6920-13-61)
Supplement: Additional file 2 — Appendix 2-A. Intra-operative technique section of the Orthopaedic Competence Assessment Project arthroscopy procedure-based assessment. Appendix 2-B: Objective Structured Assessment of Technical Skill (OSATS) Global Rating Scale. [file 1472-6920-13-61-S2.docx]

**Competencies S/U***

1 Follows an agreed, logical sequence or protocol for the

procedure

2 Consistently handles tissue well with minimal damage

3 Appropriate and safe use of instruments

4 Proceeds at appropriate pace with economy of movement

5 Deals calmly and effectively with untoward events/

complications

6 Uses assistant(s) to the best advantage at all times

7 Communicates with scrub nurse clearly and professionally

8 Clearly identifies common abnormalities such as meniscal

and ligamentous tears eg the anatomy of the torn meniscus

using hooks or probes

9 Protecting the articular surface

*S, satisfactory; U unsatisfactory

**APPENDIX 2-A**: Intra-operative technique section of the Orthopaedic

Competence Assessment Project arthroscopy procedure-based assessment

Assement is on a scale of 1 to 5 dependent on the ability of the trainee

**Score**

**Skill 1 2 3 4 5**

| 1 | Follows protocol | Unsatisfactory | Adequate. Occasional  need for guidance and  help | Excellent adherence to agreed  protocol. No prompts. No  mistakes |
| --- | --- | --- | --- | --- |
| 2 | Handles tissue  well | Careless Potential to cause  damage | Adequate. No tissue  damage. Occasional  need for increased care | Excellent tissue handling.  Precise and delicate |
| 3 | Appropriate and  safe use of instruments | Dangerous. Risk to patient  and assistant. Potential  for damage to equipment | Adequate use of instruments and scope. Occasional guidance to  ensure instruments  remain within field of  vision | Excellent use of instruments.  Good control of arthroscope.  Instruments constantly within  field of vision |
| 4 | Appropriate pace  with economy of  movement | Erratic pace and movements.  Overly rushing or  inappropriately slow | Adequate economy of  movement. Majority of  movements controlled  and careful. Occasional  erratic movement | Excellent fluidity and economy of movement. Procedure performed  at appropriate pace without  erratic movements |
| 5 | Act calmly and  effectively with  untoward events | Unable to deal with  adverse events. Panic and  inability to respond | Remains calm. Remains  safe. Takes advice from  supervisor. Unable to  cope independently | Excellent ability to cope with  adverse events. Remains calm.  Deals with complication  independently |
| 6 | Appropriate use  of assistant | Fails to involve assistant  appropriately. Resultant  poor positioning. Poor  rapport | Asks for appropriate joint position at appropriate times. Unable to suggest alternative positions  to improve view/ access | Excellent use of assistant. Good  rapport. Able to  constantly modify input of  assistant to best advantage  throughout procedure |
| 7 | Communicates  with scrub nurse | Inappropriate communication  resulting in confusion  or operative delay | Appropriate communication with scrub nurse. Occasional need for clarification from supervisor | Excellent rapport with scrub  nurse. Clear and effective  communication, maximising  procedural efficiency |
| 8 | Clearly identifies  common abnormalities | Unable to identify common  abnormalities. Confusion over basic anatomy | Adequate identification  of common pathology.  Occasional mistake. Unsure of precise classifications | Excellent knowlege of pathology of common abnormalities. Clear  understanding of classification of injuries |
| 9 | Protecting the  articular surface | Inability to protect articular  surface appropriately. Potential to cause damage | Awareness of need to  protect articular surface.  Adequate care taken. Occasional prompt from supervisor required | Excellent awarenes of articular  surfaces. High degree of care  maintained throughout the  procedure |

**APPENDIX 2-B**: Objective Structured Assessment of Technical Skill (OSATS) Global Rating Scale
